# Study on the Melt Rheological Characterization of Micro-Tube Gas-Assisted Extrusion Based on the Cross-Scale Viscoelastic Model

**DOI:** 10.3390/polym16070973

**Published:** 2024-04-03

**Authors:** Xiaohui Zhang, Xingyuan Huang, Bin Liu, Shuiquan Chen

**Affiliations:** Department of Mechanical Engineering, College of Advanced Manufacturing, Nanchang University, Nanchang 330031, China

**Keywords:** micro-tube gas-assisted extrusion, cross-scale model, DCPP model, 3D numerical analysis

## Abstract

In the micro-tube gas-assisted extrusion process, flow theories ignoring cross-scale viscoelastic variations fail to effectively characterize the rheological state of the melt. To investigate the impact of cross-scale viscoelastic variation on the quality of the micro-tube gas-assisted extrusion, a 3D multiphase flow extrusion model incorporating a double gas-assisted layer was developed. Subsequently, we modified the DCPP constitutive equations based on the cross-scale factor model. Both the traditional and gas-assisted extrusions were simulated under macroscale and cross-scale models using the Ansys Polyflow. Finally, using the established gas-assisted extrusion platform, extrusion experiments were conducted. The results indicate that, owing to the reduced melt viscosity under the cross-scale model, the deformation behavior of the melt is more pronounced than in the macroscale model. The cross-scale model’s numerical results more closely match the experimental outcomes under the same parameters, thereby confirming the feasibility of the theoretical analysis and numerical simulation. Moreover, the predictive capability of the cross-scale model for the micro-tube gas-assisted extrusion is further validated through numerical and experimental analyses with varying parameters. It is demonstrated that the cross-scale viscoelastic variation is a critical factor that cannot be overlooked in the gas-assisted extrusion.

## 1. Introduction

Polymer micro-tubes, with their small size and excellent plasticity, have been widely adopted across various industries, including the medical, telecommunications, petroleum, chemical, and automotive electronics industries [1]. Leveraging the benefits of large-scale production, extrusion has emerged as the preferred manufacturing method for polymer micro-tubes [2]. During the extrusion process, the unknown extrudate is the deformation of polymer melts out of the die as a result of the free surface flow in the air, generally referred to as the extrudate swell or die swell [3,4]. Moreover, the rheological properties of the polymer melt are significantly influenced by microscale effects due to the diminutive size of the micro-tubes during the extrusion process [5,6]. These factors directly affect the size of the micro-tubes and can lead to a compromised dimensional accuracy.

In order to suppress the extrudate swell behavior, Liang [7] first proposed gas-assisted extrusion technology in 2001. This gas-assisted extrusion technique primarily involves enhancing the extrusion die and then using a gas control system to inject gas into the interface between the die wall and the melt. This intervention induces a transition from an adherent flow to a slip flow, thereby reducing the shear stress and effectively mitigating the extrudate swell. Huang et al. [8,9] applied this gas-assisted technology to the micro-tube extrusion process. They introduced air as the assisting gas into both the inner and outer walls of the micro-tube, and their results demonstrated that the extrusion swell could be effectively suppressed.

To improve the extrusion quality of micro-tubes, the current prevailing approach involves combining numerical simulation with experimental studies to investigate the melt flow behavior and extrudate swell and then analyzing the impact of process parameters on the extrusion quality of the micro-tubes [10,11,12]. Liu B. et al. [13] simplified the physical model of the micro-tube extrusion process and subsequently established a 2D two-phase flow model based on a compressible gas and an incompressible melt. Then, the mechanism of the pressure difference between the inner and outer gas on the micro-tube extrusion quality was clarified by analyzing the distribution of the melt pressure, velocity, and stress. The results indicate that the extrudate swell of the micro-tubes is mitigated when the inner gas pressure is greater than the outer gas pressure. To investigate the extrusion swell behavior of viscoelastic polymers during the extrusion process, Liu K. et al. [14] conducted numerical simulations using the DCPP constitutive equations and developed a 3D physical model. Then, they constructed a multidimensional evaluation index to assess the extrusion quality, analyzed the velocity field and stress field to elucidate the influence of the characteristic scale on the polymer extrusion swelling behavior, and verified the correctness by extrusion experiments. Ren et al. [15] aimed to eliminate the defects of the polypropylene micro-tube during extrusion by first building a gas-assisted extrusion platform. Then, a 3D numerical simulation was conducted and the results were compared with the traditional extrusion. The results demonstrated that the gas-assisted extrusion can effectively eliminate micro-tube extrusion defects and improve smoothness and transparency. Tang et al. [16] conducted numerical simulations to investigate the differences between 3D and 2D flow models based on the multimode PTT viscoelastic model. Their study clarified that the design of extrusion dies and processes should be grounded in 3D numerical analysis. As is widely recognized, 3D flow analysis offers more intuitive and realistic results than 2D flow analysis, providing better continuity and higher scalability for current and future studies. However, in the previous micro-tube gas-assisted extrusion studies, a simplified 2D model or a simplified 3D model with complete slip conditions replacing the assisted gas are usually used. Yet, these numerical simulations have inherent limitations and fail to effectively capture the auxiliary effect of gas on the melt.

For the small die channels, the polymer characteristics transition from the macroscale to the microscale within the die, and the micro-size effect will have a certain impact on the extrudate swell during the polymer melt extrusion [17]. At the microscale, the viscoelasticity of the polymer melts decreases with a decreasing feature size, which affects the rheological behavior of the melt [18,19]. Based on the capillary model, Chien et al. [20] determined the viscosity variation rules of high-density polyethylene and polystyrene at the microscale through injection molding experiments. The results indicated that the viscosity of the solutes was reduced to varying degrees as the characteristic size of the microchannels decreased. Based on the Kelvin–Voigt ontological model, Xu et al. [21] developed a microscale viscosity to study the rheological properties of PP, PS, HDPE, and other materials under different shear rates. The results indicated that the melt viscosity decreased as the die diameter reduced at a constant shear rate. Zhao et al. [22] also investigated the effect of different micro-tube sizes on flow behavior and discovered that the rheological properties of polymers differ from those exhibited in the macroscopic state. Previous studies have established that the viscoelastic properties of the melt differ at the microscale compared to the macroscale. However, cross-scale-induced viscoelastic changes are often not accounted for in a gas-assisted extrusion numerical simulation. Therefore, to advance the development of a micro-tube gas-assisted extrusion and to enhance the extrusion precision, it is essential to investigate the mechanisms of cross-scale viscoelastic variations in a microtubule gas-assisted extrusion. In numerical studies, it is essential to choose an appropriate constitutive equation to accurately characterize the viscoelastic properties of polymer melts [23]. Initially, viscous models were commonly employed for representation. However, they proved ineffective in effectively capturing the viscoelastic properties of melts. Consequently, there is a need for constitutive equations that can capture viscoelastic phenomena, such as the PTT model [24], the Giesekus model [25,26], and the DCPP model [27]. The DCPP model overcomes certain mathematical and rheological limitations in flow simulation and provides greater insight into the extrusion swell mechanism.

Therefore, the primary objective of this study is to conduct a thorough investigation into the complex flow behavior of polymers within and outside the die during the gas-assisted extrusion, accounting for the effects of cross scale. The DCPP model, modified to incorporate a size factor, was utilized to describe the viscoelastic rheological behavior of the polymer melt within the extrusion die. Three-dimensional numerical simulations for both the traditional and gas-assisted extrusions have been carried out under macroscale and cross-scale models. Quality evaluation metrics were established to monitor the evolution of swelling behavior. A comparative analysis of velocity and stress field distributions provides insights into the potential intrinsic mechanisms. Furthermore, the validity of the cross-scale model was confirmed through numerical simulations and extrusion experiments conducted across a range of parameters.

## 2. Gas-Assisted Extrusion Modeling and Numerical Methods

### 2.1. Physical Models

**Figure 1:**
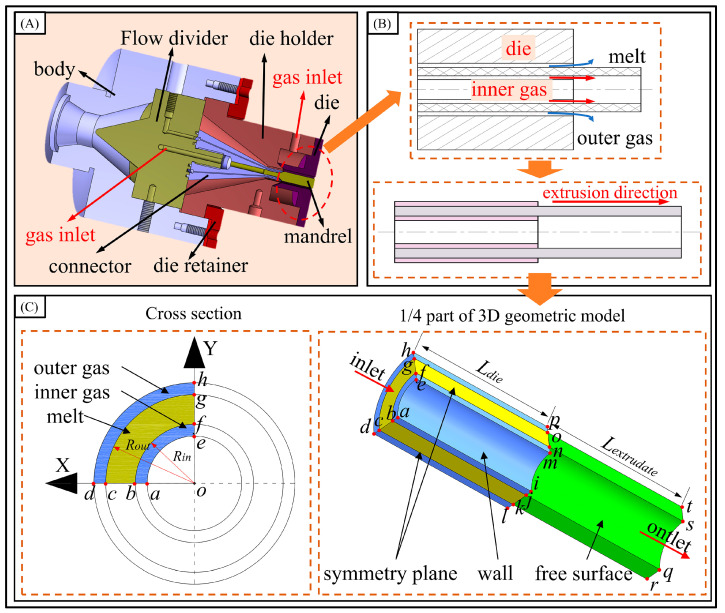
Schematic diagram of micro-tube gas-assisted extrusion model. (**A**) A 3D model of the extrusion die, (**B**) schematic diagram of fluid distribution in the gas-assisted section, and (**C**) 3D model with double-assisted gas-layer.

In this study, a finite element numerical method is employed to reveal the melt rheological behavior during the micro-tube gas-assisted extrusion under the cross-scale model. The polymer melt is heated and mixed by the extruder, then transported to the forming die where it sequentially passes through the preform, compression, and forming sections, culminating in the formation of the micro-tube. The construction process of the gas-assisted extrusion model is shown in Figure 1. Figure 1A is the 3D schematic diagram of a gas-assisted extrusion, and it can be seen that the gas-assisted forming section is mainly concentrated at the outlet. Then, by simplifying the physical model and analyzing the distribution of the melt and the gas-assisted layer, the 2D distribution diagram of fluids is obtained, as shown in Figure 1B. Given that the micro-tube features an axisymmetric geometry, a one-quarter segment of the micro-tube geometry is utilized in numerical simulations to enhance the computational efficiency. The gas-assisted extrusion 3D model with a double-assisted gas layer is shown in Figure 1C. According to the extrusion die size, the outer radius (*R*_out_) is 1.5 mm, the inner radius (*R*_in_) is 1.0 mm, and the thickness of the inner and outer gas-assisted layers is 0.2 mm. *L*_die_ represents the length of the fluid region inside the die, and *L*_die_= 10 mm. *L*_extrudate_ is the free region outside the die; to fully characterize the micro-tube extrusion swell process, it was set to 10 mm.

### 2.2. Cross-Scale Control Equations

Based on the flow characteristics of the polymer melt and gas in the die during the gas-assisted extrusion process, the following assumptions were made: since the polymer melt and gas exhibit a fully developed flow within the die and considering the melt has high viscoelasticity and low velocity while the gas has low viscosity and negligible mass, the effects of inertial force and gravity on their flow are neglected. The polymer melt is considered as an incompressible non-Newtonian fluid, and the gas is considered as a compressible Newtonian fluid [28]. Micro-tube gas-assisted extrusion is a continuous process, where the flow state follows the conservation laws. These include the laws of mass conservation, momentum conservation, and energy conservation. The conservation of mass and momentum in the gas-assisted extrusion process can be expressed by the continuity equation and motion equation, respectively.

(1) Continuity equation.

The continuity equation is derived from the law of mass conservation. Analyzing the micro-tube polymer melt and gas flow characteristics, the extrusion continuity equation is as follows:

(1) $$\frac{\partial \rho_{k}}{\partial t}+\nabla \left(\rho_{k}\cdot \nu_{k}\right)=0$$

where $\rho_{k}$ is the fluid density, $\frac{\partial \rho_{k}}{\partial t}$ is the density variation with time, $\nu_{k}$ is the fluid velocity vector, ∇ is the Hamiltonian operator, and k = I, II denote melt and gas, respectively. Because the fluid is a steady fully developed flow, the continuity equation can simplify to $\nabla \left(\nu_{k}\right)=0$.

(2) Motion equation.

The flow of melt and gas satisfies the law of momentum conservation, and the fluid within the die is in a steady state, so the motion equation is as follows:

(2) $$\rho_{k}\nu_{k}\cdot \nabla \nu_{k}+\nabla p_{k}-\nabla \cdot \tau_{k}=0$$

where $p_{k}$ is the fluid pressure and B is the fluid stress tensor. Since $\nabla \left(\nu_{k}\right)=0$, the motion equation simplifies to $\nabla p_{k}-\nabla \cdot \tau_{k}=0$.

(3) Energy equation.

The energy equation is derived from the law of energy conservation and can be specified as follows:

(3) $$\rho_{k}C_{k}(\frac{\partial T_{k}}{\partial t}+\nu_{k}\cdot \nabla T_{k})=-\nabla \cdot K-T_{k}(\frac{\partial p_{k}}{\partial T_{k}})(\nabla \cdot \nu_{k})+\tau_{k}:\nabla \nu_{k}$$

where $T_{k}$ is the fluid temperature, $C_{k}$ is the specific heat capacity at constant pressure of the fluid, K is thermal conductivity, and $\tau_{k}:\nabla \nu_{k}$ is the viscous dissipation term.

(4) Cross-scale constitutive equation.

Due to the polymer melt being a viscoelastic fluid, the DCPP viscoelastic constitutive model, which is established based on molecular theory, can provide a more accurate representation of melt viscoelastic rheological properties. Additionally, it can predict the impact of shear thinning and the normal stress difference on the elastic recovery of the melt. It can also can reveal the extrusion intrinsic mechanism from the perspective of the molecular chain movement. Consequently, the DCPP model is employed to describe the melt flow. The DCPP model is as follows [29]:

(4) $$\tau_{Ι}=\tau_{Ι}^{E}+\tau_{Ι}^{V}$$

(5) $$\tau_{Ι}^{E}=\frac{G}{1-\xi }\left(3\Lambda^{2}S-I\right)$$

(6) $$\lambda \left[(1-\frac{\xi }{2})\overset{\nabla }{S}+\frac{\xi }{2}\overset{\Delta }{S}\right]+\lambda (1-\xi )\left[2D:S\right]S+\frac{1}{\Lambda^{2}}\left[S-\frac{I}{3}\right]=0$$

(7) $$\lambda_{s}\frac{D\Lambda }{Dt}-\lambda_{s}(D:S)\Lambda +(\Lambda -1)e^{\frac{2(\Lambda -1)}{q}}=0$$

where $\tau_{Ι}^{E}$ is the viscoelastic extra stress tensor and $\tau_{Ι}^{V}$ is the purely viscous component. For $\tau_{Ι}^{V}=2\eta_{h}D$, $\eta_{h}$ is the Newtonian contribution viscosity and *D* is the rate of the deformation tensor, which is related to the velocity gradient. *G* is the melt shear modulus; ξ is the dimensionless coefficient controlling the second normal stress differential coefficient; S and Λ are the state variables, the orientation tensor, and the stretching scalar, respectively; and I is defined as the unit tensor. The superscripts ∇ and Δ are the upper and lower convected derivatives, respectively. λ is the orientation relaxation time; $\lambda_{s}$ is the tensile relaxation time; and q is the number of end-branches in the main chain of the molecule, which control the transient tensile properties of the material.

The microscale viscosity model can respond to changes in the characteristics of the melt flow in the microchannel to a certain extent. Xu Bin et al. [21] established a microscale viscosity model based on the Kelvin–Voigt intrinsic equation, demonstrating that the melt viscosity decreases as the characteristic size is reduced. To more accurately describe the micro-tube extrusion process based on the macroscale viscosity model, the micro-size factor is introduced to correct the melt viscosity model and to obtain the cross-scale viscosity equation, as follows:

(8) $$\tau_{Ι}^{V}=2\eta_{w}D=2\eta_{h}[1.1-{\left(\alpha \cdot \frac{1}{B}\right)}^{\beta }/\gamma ]D$$

where $\eta_{w}$ is the melt viscosity considering the microscale; *B* is the feature size, which can be represented by the channel thickness, i.e., *B* = *R*_out_ − *R*_in_; and α, β, and γ are the material modelling coefficients.

(5) Gas state equation.

In the simplified model, the gas is considered a compressible Newtonian fluid. To ensure the closed-loop nature of the numerical solution equations set for the gas, the gas state equation is used to describe the gas flow process as follows:

(9) P=ρRT

where *P* is the gas pressure; ρ is the gas density; *T* is the gas temperature; and *R* is the gas constant, where R = 8.314 J/(mol·K).

### 2.3. Boundary Conditions

The polymer melt is extruded along the *z*-axis under the influence of the gas, resulting in variations in velocity and stress in both the radial and axial directions. To define the boundary conditions, *f*_n_ and *f*_s_ are used to represent the normal and tangential stresses of the fluid. *v*_n_ and vs. denote the normal and tangential velocities. According to the gas-assisted extrusion geometrical model constructed in Figure 1, the boundary conditions are set as detailed in Table 1.

(1) Melt inlet boundary: Due to the assumption that the polymer melt flows as a fully developed non-Newtonian viscoelastic flow when it enters the shaping section, its velocity along the extrusion direction is constant and the radial velocity is 0. Therefore, the melt satisfies the following relation at the inlet interface: $\partial \nu_{z}/\partial z=0,\nu_{x}=\nu_{y}=0$, where *ν*_x_, *ν*_y_, and *ν*_z_ are the velocities of the melt in the x, y, and z directions, respectively. Thus, the melt inlet boundary is defined as the flow inlet boundary with a flow rate set at 5 mm^3^/s.
(2) Gas inlet boundary: After the gas enters its form as a stable gas cushion layer, the gas is in a fully developed state at this time. As a result, the gas inlet is set to meet the relationship $\partial \nu_{z}/\partial z=0,\nu_{x}=\nu_{y}=0$ and the inlet interface mechanics relationship to meet $f_{s}=0$. Therefore, in the numerical simulation, the gas inlet is set as the pressure inlet boundary conditions. Since the inner gas pressure needs to be greater than the outer gas pressure [13], we set the inner gas pressure to 6000 Pa and the outer gas pressure to 5000 Pa initially.
(3) Wall boundary: In the gas-assisted extrusion process, the gas forms a stable gas cushion layer, so the melt is not in contact with the die wall but with the gas. There is almost no relative slip between the gas and the wall, so the wall adopts the complete slip boundary condition, which satisfies the relation $\nu_{n}=0,f_{s}=0$.
(4) Interface boundary: At the interface between the gas and the melt, the relative slip and surface tension between the gas and melt are neglected, the stresses on both sides of the interface are in equilibrium, and the fluid does not penetrate the interface. That is, the boundary satisfies the dynamics condition $f_{s}^{I}=f_{s}^{\mathrm{II}},f_{n}^{I}=f_{n}^{\mathrm{II}},\nu_{s}^{I}=\nu_{s}^{\mathrm{II}}$, where I and II denote the melt and gas, respectively.
(5) Free surface boundary: After the polymer melt is extruded out of the die, in the absence of external pressure and surface tension on the inner and outer walls, the boundary satisfies the following dynamics conditions: $f_{n}=f_{s}=0,\nu_{n}=0$.
(6) Symmetry plane boundary: To improve the computational efficiency of the numerical simulation, the one-quarters micro-tube model is used for the simulation, so there are two symmetry planes that are not affected by the tangential force and normal velocity, and these meet the following boundary conditions: $f_{s}=0,\nu_{n}=0$.
(7) Melt outlet boundary: Without any traction force applied to the end of the micro-tube melt, its end is sufficiently cooled and shaped so that the following boundary conditions are satisfied: $f_{n}=0,\nu_{s}=0$.
(8) Gas outlet boundary: Due to the unknown pressure and motion state of the gas at the die outlet, the outflow condition is set, satisfying the following dynamics conditions: $f_{s}=0,\partial \nu_{n}/\partial t=0$.

### 2.4. Numerical Simulation Parameters

The polymer utilized in this study is polypropylene. According to the DCPP constitutive model, it is necessary to determine the five material parameters, the Newtonian contribution viscosity of the polymer, and the model coefficients for the size factor. The parameters of the four-modal DCPP constitutive model for polypropylene are shown in Table 2 [30]. According to the study by Wang et al. [31] about the microscale viscosity measurement and the viscosity modelling of polymers, the Newtonian contribution viscosity is set to 6997 Pa·s, and α, β, and γ are 13.1265, 0.9084, and 96.8086, respectively. The size factor *B*, which uses the average wall thickness, is set to 0.5 mm. In our experiments, we used air as the auxiliary gas; being a Newtonian fluid, air maintains a stable viscosity of 2.6 × 10^−5^ Pa·s.

### 2.5. Quantification of Extrusion Quality

To systematically analyze the deformation phenomena of micro-tubes during the gas-assisted extrusion process, quality analysis indices are established to quantitatively analyze the parison deformation behaviors, including the inner radius, outer radius, wall thickness, and cross-sectional area swell rate. The evaluation indicator formula is as follows:

(10) $$W_{in}=\frac{R_{in-E}}{R_{in-D}}\times 100\%$$

(11) $$W_{out}=\frac{R_{out-E}}{R_{out-D}}\times 100\%$$

(12) $$W_{wall}=\frac{\left(R_{out-E}-R_{in-E}\right)}{R_{out-D}-R_{in-D}}\times 100\%$$

(13) $$W_{S}=\frac{\left({R^{2}}_{out-E}-{R^{2}}_{in-E}\right)}{{R^{2}}_{out-D}-{R^{2}}_{in-D}}\times 100\%$$

where *W*_in_, *W*_out_, *W*_wall_, and *W*_S_ represent the inner radius, outer radius, wall thickness, and cross-sectional area swell rate, respectively. *R*_in-E_ and *R*_out-E_ represent the inner radius and outer radius of the micro-tube. *R*_in-D_ and *R*_out-D_ represent the inner radius and outer radius of the die.

### 2.6. Numerical Calculation

To ensure a stable solution for the 3D viscoelastic extrusion of the micro-tubes, this study employs a numerical approach that combines EVSS, SU, and Mini-element to solve the stress field and velocity field in the finite element calculation. Additionally, a low interpolation method is used, which couples the quadratic velocity and linear pressure, in order to optimize the computation time. The free surface is handled using the Streamwise method to reset the free surface mesh outside the die. Given the highly nonlinear rheological behavior of viscoelastic fluids, the motion boundaries are defined using an evolutionary method to ensure convergence of the calculations. Furthermore, the Galerkin’s method is employed in the iterative calculations.

## 3. Numerical Results and Analysis

### 3.1. Extrusion Quality Results and Analysis

**Figure 2:**
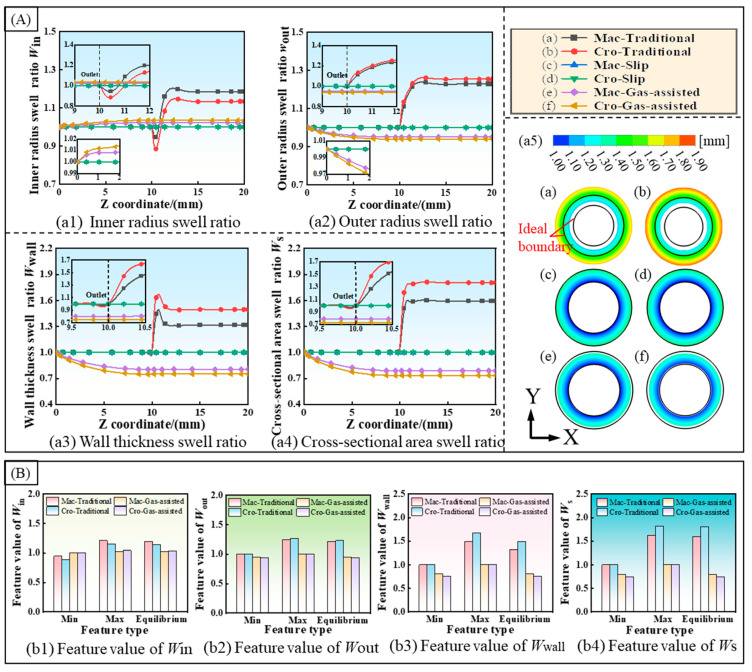
Numerical extrusion size and feature values of micro-tube. (**A**) Size swell ratio distribution: (**a1**) inner radius swell ratio, (**a2**) outer radius swell ratio, (**a3**) wall thickness swell ratio, (**a4**) cross-sectional area swell ratio, and (**a5**) radius distribution at die outlet; and (**B**) feature values distribution. (**b1**) feature value of *W*_in_, (**b2**) feature value of *W*_out_, (**b3**) feature value of *W*_wall_, (**b4**) feature value of *W*_s_.

To investigate the effect of cross-scale viscosity changes on the extrusion quality and to validate the gas-assisted extrusion 3D model, macroscale and cross-scale numerical simulations were conducted for three models: traditional extrusion, gas-assisted extrusion with slip boundary conditions, and gas-assisted extrusion with a double-assisted gas-layer. The swelling behavior of the micro-tube along the extrusion direction, in terms of the inner radius, outer radius, wall thickness, and cross-sectional area, was analyzed, and the results are presented in Figure 2. The prediction curves for the four evaluation indicators are shown in Figure 2A. When comparing the variation curves of the inner radius swell ratio, *W*_in_, in the traditional extrusion, there is an instantaneous decrease in *W*_in_ and *W*_in_ < 1 as the melt leaves the die. This is followed by an instantaneous increase to a maximum value over a short distance and it eventually reaching an equilibrium value after a gradual decrease. The rate of change of *W*_in_ in the cross-scale model is smaller than in the macroscale model. In the case of the complete slip boundary extrusion, the inner radius swell rate remains constant from the inlet to the outlet, with *W*_in_ = 1. In the double gas-layer gas-assisted extrusion model, *W*_in_ increases slowly from the melt inlet and reaches a steady value before leaving the die. The variation of the inner radius under the cross-scale model is slightly larger than the macroscale model, contrary to the traditional extrusion. In the outer radius swell rate curve, the change differs from the inner radius in the traditional extrusion: it directly and rapidly increases to the maximum value, then slowly declines to a stable value. The outer radius variation under the cross-scale model is larger than in the macroscale. In the slip boundary model, the outer radius swell rate remains constant, the same as the inner radius, with *W*_out_ = 1. In the double gas-layer gas-assisted extrusion model, the outer radius swell rate starts decreasing from the inlet and reaches the equilibrium value before the die outlet. The variation under the microscale model is larger than in the macroscale. While the wall thickness variation differs from that of the inner radius and outer radius, its variation is concentrated within a short distance. In the traditional extrusion model, the wall thickness variation increases rapidly to a maximum value within a short distance from the die exit, then decreases rapidly to a stable value. In contrast, in the double gas-layer gas-assisted model, the wall thickness decreases rapidly from the inlet and reaches the equilibrium value. The trend of the cross-sectional area change is similar to that of the wall thickness. It can be observed in the cross-sectional dimensional clouds after the stabilization of the micro-tube extrusion that the traditional extrusion exhibits obvious swelling behavior, while, under the slip boundary, the extrusion boundary contour perfectly coincides with the theoretical contour. In contrast, the double gas-layer gas-assisted extrusion exhibits noticeable shrinkage. Under the slip boundary conditions, the melt flow achieves an ideal, steady state, and the extrusion dimensions perfectly correspond to the theoretical predictions, although these might not align with actual production scenarios. Consequently, the main focus of the next study is to analyze the traditional extrusion and double gas-layer gas-assisted extrusion models.

To characterize the melt extrusion shrinkage behavior, ultimate deformation, and steady state, the minimum, maximum, and equilibrium values of the variation curves are illustrated in Figure 2B. Observations reveal that the cross-scale model exerts a greater influence on the traditional extrusion process compared to the gas-assisted extrusion, with a more pronounced effect on the wall thickness and cross-sectional area. During the air-assisted extrusion, the peak values are similar, although the variation of the cross-scale model is slightly larger than that of the macroscale. This indicates that variations in the melt cross-scale viscosity cannot be neglected during the gas-assisted extrusion.

### 3.2. Velocity Distribution Results and Analysis

**Figure 3:**
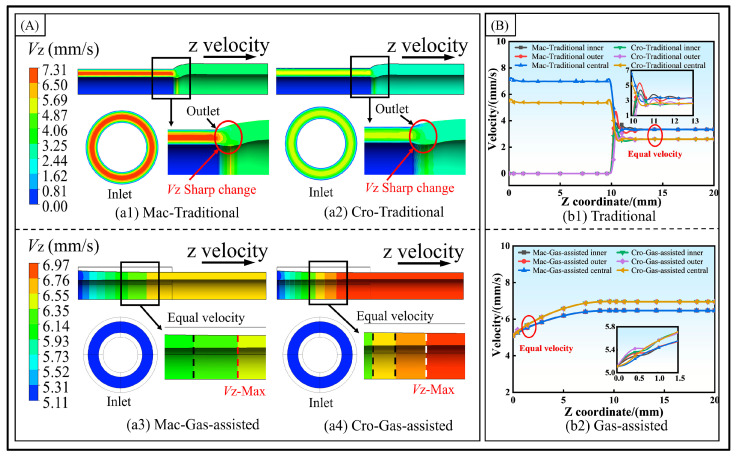
Axial velocity distribution. (**A**) Axial velocity cloud diagram, and (**B**) axial velocity of inner and outer wall.

**Figure 4:**
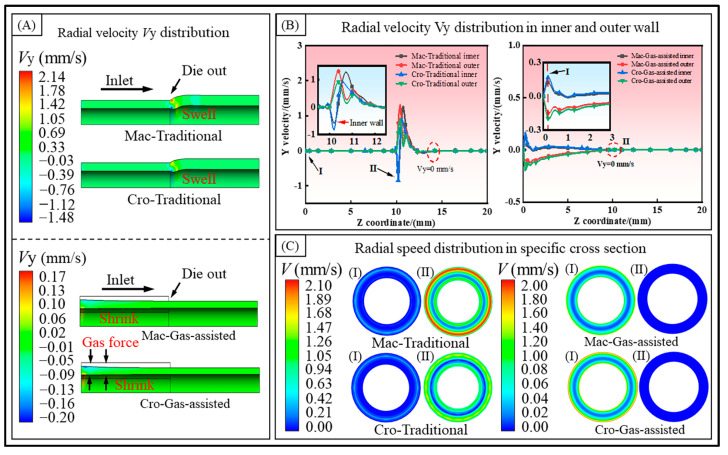
Radial velocity distribution. (**A**) Radial velocity cloud diagram, (**B**) radial velocity of inner and outer walls, and (**C**) radial speed in specific cross section.

As depicted in Figure 3 and Figure 4, the distributions of the melt velocity field under different models are utilized to reveal the deformation behavior attributable to the melt’s rheological properties during the micro-tube extrusion at the cross-scale.

(1) Axial velocity.

The axial velocity distribution of the melt is shown in Figure 3A. In the traditional extrusion model, due to the non-slip wall condition adopted on the inner wall surface of the die, the velocity distribution is low on both sides and high on the inner side. After reaching the die outlet, the velocity reaches equal velocities in a short distance. The velocity extremes under the cross-scale model are smaller than those under the macroscale. In the gas-assisted extrusion, the axial velocity quickly reaches a consistent value and then gradually increases to a maximum value before remaining constant. This occurs because the gas has an axial drag effect on the melt, which increases the melt axial velocity. With a constant inlet velocity, this leads to melt stretching and shrinkage, thereby explaining the wall thickness shrinkage phenomenon observed under the gas-assisted model. Figure 3B shows the velocity variations at the inner wall, outer wall, and midline of the melt. In the traditional extrusion, the velocities of these regions reach equality slowly after the die inlet. In the gas-assisted model, their velocities reach unity around 1 mm into the die, gradually increase, and remain unchanged after the die outlet. Interestingly, observations indicate that under the traditional model, the velocity within the cross-scale framework is lower than that in the macroscale model. Conversely, in the gas-assisted model, the velocity in the cross-scale model is larger than in the macroscale model. This is because the melt viscosity decreases under the cross-scale model. In the traditional model, the viscous effect at the inner and outer walls is smaller than in the macroscale model, which results in a larger effective flow range and, consequently, a lower velocity. In the gas-assisted model, the response to the gas drag effect is more pronounced, leading to a greater velocity.

(2) Radial velocity.

Figure 4 illustrates the radial velocity distribution of the melt and the velocity variation curves of the inner and outer walls. As shown in Figure 4A, the radial velocity distribution is depicted. In the traditional extrusion, the radial velocity increases rapidly at the die outlet, and both the inner and outer walls exhibit a speed that expands outward. However, the inner wall velocity rapidly increases from negative to positive values, then aligns with the direction of motion of the outer wall surface and quickly reaches a maximum value. Both velocities decrease to zero at different rates. The negative value of the inner wall velocity also accounts for the transient shrinkage observed at the die outlet. In the gas-assisted extrusion, a certain radial velocity directed inward is observed at the die inlet. The melt tends to flow inward, which explains the shrinkage of the melt wall thickness. From the radial velocity curves of the inner and outer walls of the melt shown in Figure 4B, it can be seen that in the traditional extrusion, the inner wall velocity under the cross-scale model is smaller than that of the macroscale model, and the distance over which both velocities drop to zero is equal. According to the integral theory, the distance of the outward movement of the inner wall in the cross-scale model is smaller than that in the macroscale model. This explains why the swell rate of the inner radius is smaller in the cross-scale model than in the macroscale model, as shown in Figure 2A. Conversely, the situation for the outer wall surface is exactly the opposite. In the gas-assisted extrusion model, the inward motion of both the inner and outer walls of the cross-scale model is greater than in the macroscale model, and both drop to zero near the exit. This also explains why the variations in the inner radius, outer radius, wall thickness, and cross-sectional area are greater in the cross-scale model. In Figure 4C, we depict the velocity distribution of the microtubule cross section at the two characteristic point locations identified in Figure 4B. The melt radial velocities are primarily concentrated at the inner and outer walls. Moreover, in the gas-assisted model, the melt radial velocities achieve equilibrium within the die and remain unchanged after exiting the die.

### 3.3. Pressure Distribution Results and Analysis

**Figure 5:**
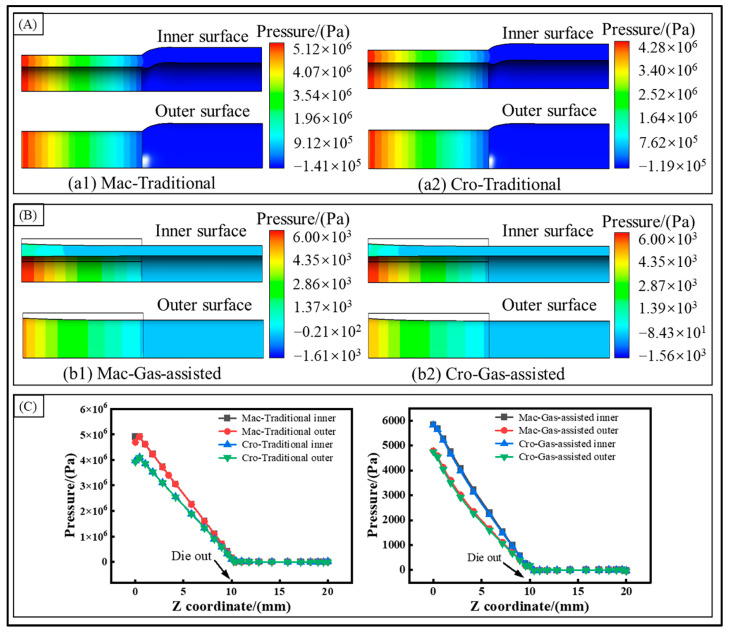
Pressure distribution. (**A**) Pressure distribution of traditional extrusion, (**B**) pressure distribution of gas-assisted extrusion, and (**C**) pressure distribution of inner and outer walls.

To better understand the impact of the cross-scale model on the quality of the micro-tube extrusion, the distribution of the melt pressure was analyzed, as depicted in Figure 5. In the traditional extrusion, the melt pressure decreases linearly along the extrusion direction. The pressure value of the melt under the cross-scale model is smaller than that under the macroscale model. This is because the melt viscosity in the macroscale model is greater than in the cross-scale model, leading to a larger viscous effect on the die’s inner and outer walls, and the effective extrusion range is smaller. With the flow rate remaining constant, this leads to higher pressure. This further explains the large axial velocity of the melt. In the gas-assisted extrusion, the change in the melt pressure is nonlinear, and the pressure drop is mainly concentrated in the front half of the die, falling to zero before the melt leaves the die. When comparing the traditional extrusion with the gas-assisted extrusion, the inner and outer boundaries of the die approximate a slip boundary with gas assistance. The viscous effect of the melt is reduced; thus, the melt extrusion pressure is lowered, and the swelling behavior of the micro-tubes is greatly diminished.

### 3.4. Shear Rate Distribution Results and Analysis

**Figure 6:**
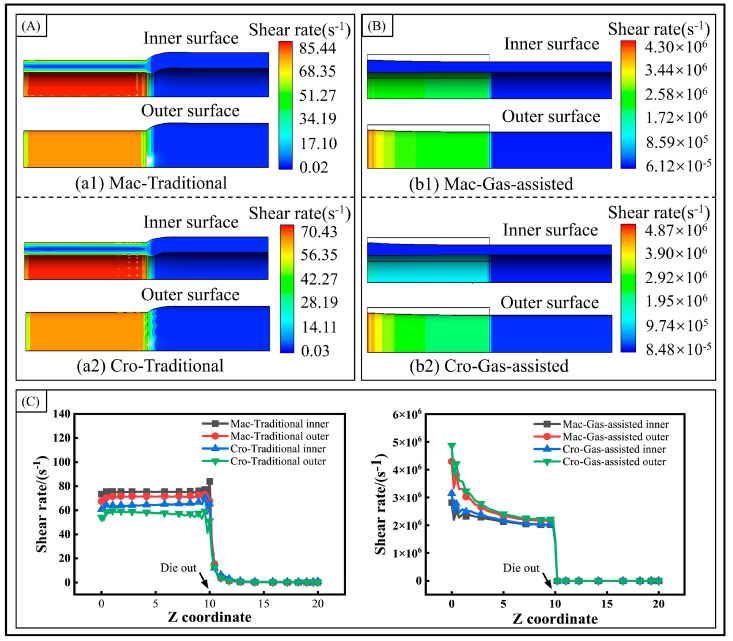
Shear rate distribution. (**A**) Shear rate distribution of traditional extrusion, (**B**) shear rate distribution of gas-assisted extrusion, and (**C**) shear rate distribution of inner and outer walls.

The shear rate distribution of the extrusion process is depicted in Figure 6. In the gas-assisted extrusion, the melt surface undergoes a significantly higher shear rate compared to the traditional extrusion. However, the shear rate is primarily distributed on the melt surface and rapidly decreases to zero upon exiting the die outlet. This indicates that the melt surface is significantly dragged by the gas. In contrast, in the traditional extrusion, the shear rate distribution extends throughout the melt interior and gradually drops to zero over a certain distance after leaving the die, thereby causing the extrusion swell phenomenon. In the cross-scale model, the shear rate in the traditional extrusion is smaller than in the macroscale model. Conversely, in the gas-assisted extrusion, the shear rate is larger than in the macroscale model, due to the reduced viscoelasticity and the more pronounced response to the gas action. This also explains the more drastic deformation behavior of the melt at the cross-scale. To analyze the surface shear rate distribution more clearly, the inner and outer surface shear rate distribution curves were obtained, as shown in Figure 6C. In the traditional extrusion, a sudden increase in the shear rate occurs at the die outlet, which increases the melt’s elastic energy storage and tensile stress, thereby exacerbating the extrusion swell and extrusion fracture phenomena. In the gas-assisted extrusion, the shear rate decreases gradually, and, upon leaving the die, it loses the gas effect and drops to zero almost instantaneously, preventing further deformation of the melt after it exits the die.

### 3.5. First Normal Stress Difference Distribution Results and Analysis

**Figure 7:**
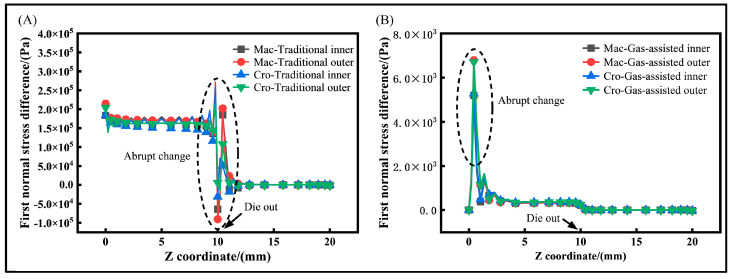
First normal stress difference distribution. (**A**) First normal stress difference distribution of traditional extrusion, and (**B**) first normal stress difference distribution of gas-assisted extrusion.

During the extrusion process of the micro-tube, the first normal stress difference of the molten material directly influences its swelling behavior. The distribution curve of the first normal stress difference for the inner and outer wall surfaces is shown in Figure 7. In the traditional extrusion, the first normal stress difference within the die is much larger than in the gas-assisted extrusion. It initially increases and then decreases, exhibiting an oscillatory curve that diminishes to zero over a short distance. This is because a larger first normal stress difference enhances the tendency of the melt to swell. Such a trend also exacerbates the swell behavior at the die exit in the traditional model. In the gas-assisted extrusion, a significant first normal stress difference is observed only near the die inlet, after which it gradually decreases and drops to zero at the die outlet. In the cross-scale model, the first normal stress difference in the traditional extrusion is slightly smaller than in the macroscale model, while the opposite is true for the gas-assisted extrusion. This is primarily due to the melt viscosity being reduced under the cross-scale model. The numerical results indicate that the gas-assisted extrusion can effectively mitigate the swell behavior, and changes in viscosity under the cross-scale model directly impact the forming quality of the micro-tube.

## 4. Numerical Simulation with Different Parameters

### 4.1. The Effect of Gas Pressure in Gas-Assisted Extrusion

**Figure 8:**
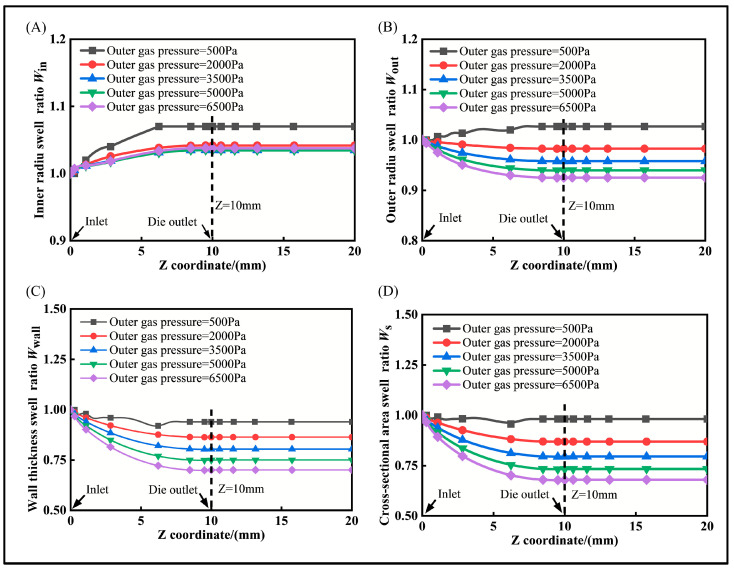
The effect of gas pressure in gas-assisted extrusion. (**A**) Inner radius swell ratio distribution, (**B**) outer radius swell ratio distribution, (**C**) wall thickness swell ratio distribution, and (**D**) cross-sectional area swell ratio distribution.

To investigate the effect of gas pressure on the micro-tube swell behavior, while keeping the inner and outer pressure difference and other conditions constant, the swell behavior was examined. The swell rates of the micro-tube’s inner radius, outer radius, wall thickness, and cross-sectional area along the extrusion direction at various pressures are depicted in Figure 8.

It is clear that the micro-tube deformation is fully completed within the die, and the swell ratio remains constant upon exiting the die outlet. With increasing pressure, the melt shrinkage behavior intensifies, leading to a thinner wall thickness. However, if the pressure is too low, a stable gas cushion layer cannot form. Therefore, strict control of the gas pressure is essential to achieve micro-tubes with a high extrusion quality.

As the gas pressure increases, the inner radius of the micro-tube expands, but the extent of this expansion decreases significantly. The outer radius exhibits a negative expansion phenomenon, with *W*_out_ < 1, indicating a reduction in the outer radius and an inward movement. As a result, the wall thickness and cross-sectional area decrease. It is evident that the micro-tube deformation is fully completed within the die, and the swell ratio remains constant upon exiting the die outlet. With increasing pressure, the melt shrinkage behavior intensifies, and the wall thickness becomes thinner. However, if the pressure is too low, a stable gas cushion layer cannot form. Therefore, strict control of the gas pressure is essential to achieve micro-tubes with a high extrusion quality.

### 4.2. The Effect of Inner and Outer Gas Pressure Difference in Gas-Assisted Extrusion

**Figure 9:**
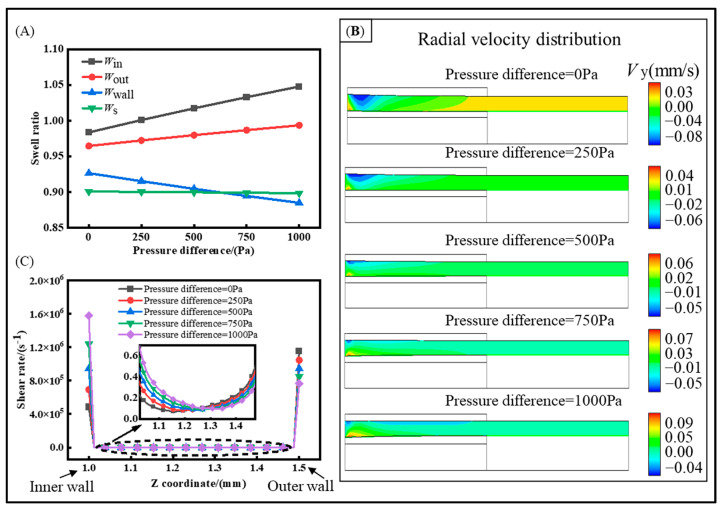
The effect of inner and outer gas pressure difference. (**A**) Swell ratio distribution, (**B**) radial velocity distribution, and (**C**) shear rate distribution of inlet.

Based on the findings in Section 4.1, the deformation of the outer radius is minimized at a gas pressure of 2000 Pa. However, the inner radius swells at a higher rate due to excessive inner gas pressure. In order to improve the quality, we investigated how the differences in the inner and outer pressures affect the swell behavior of the melt. The extrusion quality at pressure differences of 0, 250, 500, 750, and 1000 Pa with a constant outer gas pressure of 2000 Pa was primarily investigated, and the results are shown in Figure 9. As the pressure difference increases, the swell rate of the inner radius exceeds that of the outer radius, and the change in the outer radius gradually approaches the ideal value. However, the difference between the inner and outer radii gradually increases. Additionally, the wall thickness becomes thinner with the increase in differential pressure. It can be observed in Figure 9B that, with the increase in the pressure difference, the melt’s inward velocity is greater, which exacerbates the melt shrinkage phenomenon. In the shear rate distribution shown in Figure 9C, an increase in the inner pressure leads to an increase in the shear rate at the inner surface and a decrease in the shear rate at the outer surface. However, when there is no pressure difference between the inner and outer surfaces, the shear rate at the inner surface is lower than that at the outer surface.

## 5. Experimental Results and Analysis

### 5.1. Experimental Equipment

**Figure 10:**
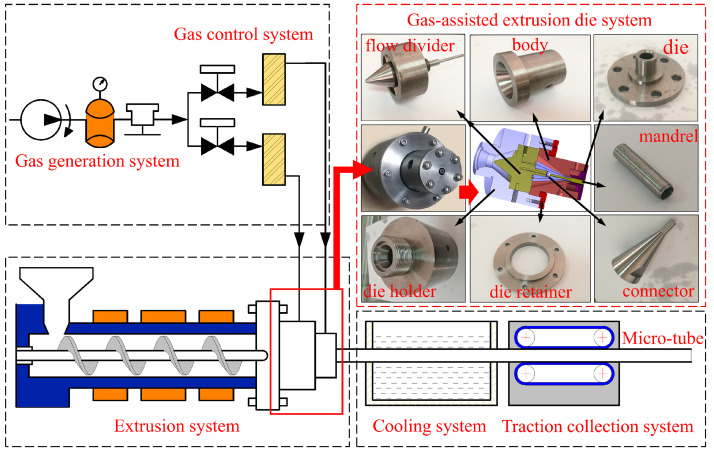
The schematic diagram of gas-assisted extrusion system.

To verify the accuracy of the numerical results, a micro-tube gas-assisted extrusion system was constructed, as depicted in Figure 10. The extrusion system primarily comprises an extrusion system, a cooling system, a traction collection system, a gas-assisted extrusion die system, a gas generation system, and a gas control system. The extrusion raw material utilized is polypropylene (PPH-T03, produced by Beijing Sinopec Corporation, with a melting point of 164 °C and a density of 900 kg/m^3^). The polymer pellets are melted and mixed by the extruder, aided by various systems, and ultimately formed into micro-tubes. Subsequently, different processing parameters are set to examine their impact on the extrusion quality of the micro-tubes. The conditions of melt inlet flow rate, traction speed, and melt temperature are consistent between the gas-assisted extrusion experiments and the traditional extrusion experiments, as shown in Table 3.

### 5.2. Extrusion Quality Results and Analysis

**Figure 11:**
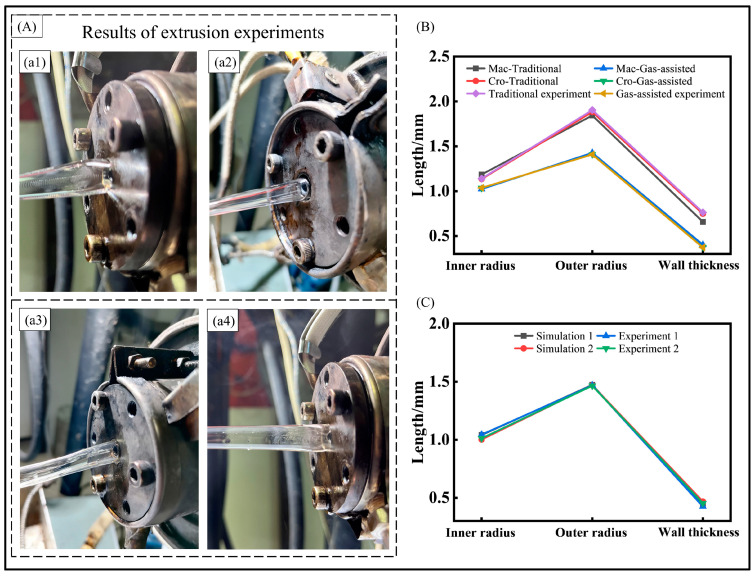
Extrusion Experiment results. (**A**) Result of the micro-tube extrusion experiment. (**a1**) extrusion results of the traditional extrusion, (**a2**) extrusion results of the gas-assisted extrusion, (**a3**) result of the gas-assisted extrusion with an outer pressure of 2000 Pa, (**a4**) result of the gas-assisted extrusion when the pressure difference is 250 Pa; (**B**) size comparison of numerical and extrusion experiments under the initial parameters, and (**C**) the simulation and experimental results for the two sets of parameters in Section 4.1 and Section 4.2.

To verify the numerical results, we conducted four extrusion experiments with different parameters. The extrusion experimental results are presented in Figure 11. Figure 11A displays the results of the micro-tube extrusion experiment. Specifically, (a1) and (a2) correspond to the results of the traditional extrusion and gas-assisted extrusion using the initial parameters, respectively. Additionally, (a3) shows the result of the gas-assisted extrusion with an outer pressure of 2000 Pa, as discussed in Section 4.1. Furthermore, (a4) illustrates the result of the gas-assisted extrusion when the pressure difference is 250 Pa, as explored in Section 4.2.

In the traditional extrusion, a significant extrusion swell behavior is observed at the die outlet, followed by shrinkage within a short distance. This observation is consistent with the numerical results depicted in Figure 2. In contrast, the gas-assisted extrusion features an evident gas cushion layer between the micro-tube and the die walls. Moreover, as the process parameters are refined, the shrinkage behavior of the micro-tube is effectively mitigated, and the evaluation indices of the micro-tube more closely align with theoretical values. This validation of the effectiveness of the gas-assisted extrusion suggests that it can significantly reduce the extrusion swell behavior.

Figure 11B presents the numerical and experimental results for the micro-tubes’ inner radius, outer radius, and wall thickness under the initial parameters. In the conventional extrusion experiments, the inner radius, outer radius, and wall thickness measure 1.14 mm, 1.90 mm, and 0.76 mm, respectively. For the gas-assisted extrusion experiment, these dimensions are 1.04 mm, 1.41 mm, and 0.37 mm, respectively. Observations indicate that the experimental outcomes closely align with the numerical simulation results for both the traditional and gas-assisted extrusions under the cross-scale model. Further analysis of the simulation and experimental results for the two sets of parameters discussed in Section 4.1 and Section 4.2 further confirms the accuracy of the numerical results under the cross-scale model, as depicted in Figure 11C. The variation in the melt viscosity under the cross-scale model is a significant factor that cannot be overlooked during the micro-tube gas-assisted extrusion.

## 6. Conclusions

(1) To investigate the impact of melt viscosity variation on the micro-tube extrusion quality, a multiphase flow gas-assisted extrusion 3D model was constructed. The size factor was then introduced to formulate the cross-scale viscosity equation, and the DCPP viscoelastic constitutive model was refined. Subsequently, a multidimensional extrusion quality evaluation system was established. Ultimately, the numerical and experimental results from the traditional extrusion and gas-assisted extrusion under both the macroscale and cross-scale models were compared. The melt deformation behavior under the cross-scale model is more pronounced than under the macroscale. Furthermore, the numerical results under the cross-scale model align more closely with the experimental outcomes, thereby validating the feasibility of the theoretical analysis and numerical simulation. The predictive capability of the cross-scale model is further confirmed through numerical simulations with varied parameterizations and experimental analysis. It was demonstrated that the cross-scale effect is critical and cannot be overlooked in the design of the micro-tube extrusion dies and in the numerical simulation.
(2) Furthermore, to better control the gas pressure, the effects of the gas pressure and gas pressure differences were investigated. As the gas pressure increases, the inner radius tends to increase and the outer radius tends to decrease, implying that the shrinkage behavior becomes more pronounced; however, the rate of change slows down. With an increase in the pressure difference, the change rate of the inner radius accelerates, while the change rate of the outer radius decelerates, and the center line moves outward. This suggests that an appropriate pressure difference can effectively minimize the discrepancies between the inner and outer dimensions, thereby reducing any dimensional deformation.
(3) Due to the interactions among the process parameters, the coupled effects of the extrusion flow rate, gas pressure, pressure difference, temperature, and viscoelastic behavior will be further considered in future studies. This approach aims to more accurately reflect the actual extrusion process and to develop reasonable extrusion parameters.

## Figures and Tables

**Table 1:** Boundary conditions.

	Type of Boundary Conditions	Faces	Boundary Types	Boundary Conditions
1	Melt inlet boundary	bcgf	Volume flow inlet	$\partial \nu_{z}/\partial z=0,\nu_{x}=\nu_{y}=0$
2	Gas inlet boundary	abfe, cdhg	Pressure inlet	$f_{n}=C,f_{s}=0$
3	Wall boundary	dhpl	Full slip condition	$\nu_{n}=0,f_{s}=0$
4	Interface boundary	cgok, bfnj	Interface boundary	$f_{s}^{I}=f_{s}^{\mathrm{II}},f_{n}^{I}=f_{n}^{\mathrm{II}},\nu_{s}^{I}=\nu_{s}^{\mathrm{II}}$
5	Free surface boundary	Jnsq, kotr	Without forces	$f_{n}=f_{s}=0,\nu_{n}=0$
6	Symmetry plane	ehts, adrq	Plane of symmetry	$f_{s}=0,\nu_{n}=0$
7	Melt outlet boundary	qrts	Without traction forces	$f_{n}=0,\nu_{s}=0$
8	Gas outlet boundary	Ijnm, klpo	Outflow	$f_{s}=0,\partial \nu_{n}/\partial t=0$

**Table 2:** Material parameters of the multi-mode DCPP model.

Mode	G/(Pa)	λ/(s)	$\lambda_{s}$/(s)	q	ξ
1	334.3	6.317	3.158	8	0.105
2	5236	0.5471	0.2736	4	0.125
3	30990	0.052	0.026	1	0.2
4	97460	0.0042	0.0021	1	0.2

**Table 3:** Experimental conditions.

Experiment Conditions	Gas-Assisted Extrusion	Traditional Extrusion
Temperature of the die (°C)	215	215
Extruder speed (r/min)	5	5
Temperature of the gas (°C)	210	/
Temperature of the melt (°C)	215	215
Pulling speed (r/min)	4	4

## Data Availability

Data are contained within the article.
